# Supplementary material for: Raman spectroscopic signature of fractionalized excitations in the harmonic-honeycomb iridates β- and γ-Li2IrO3
Source: Nat Commun. 2016 Jul 26;7:12286. doi: 10.1038/ncomms12286 (PMC4963532; doi:10.1038/ncomms12286)
Supplement: Supplementary Information — Supplementary Figures 1-3, Supplementary Tables 1-2, Supplementary Notes 1-4 and Supplementary References [file ncomms12286-s1.pdf]

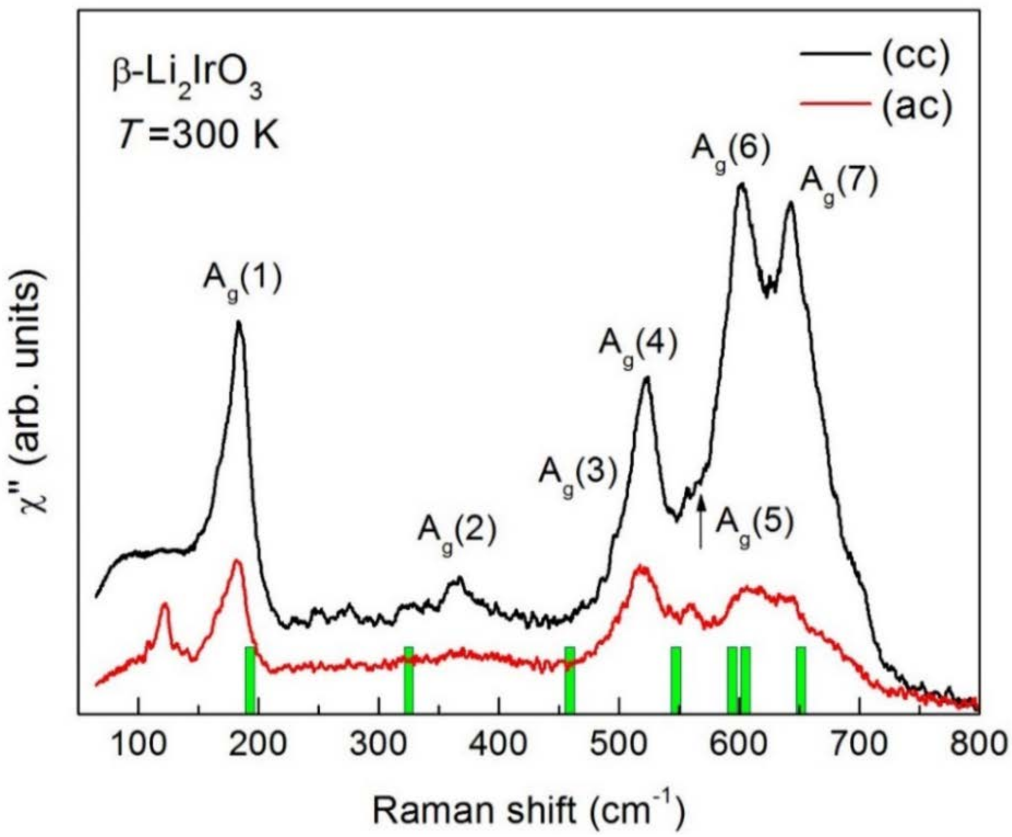

Supplementary Figure 1 | Comparison of Raman spectra of β-Li_2IrO_3 measured at *T*=300 K in (*cc*) and (*ac*) polarization. Seven A_g modes are assigned according to the lattice dynamical calculations made using interionic potentials (see Supplementary Table 1). The calculated frequencies are marked by green bars. See Supplementary Note 1 and Supplementary Table 2 for detailed discussions.

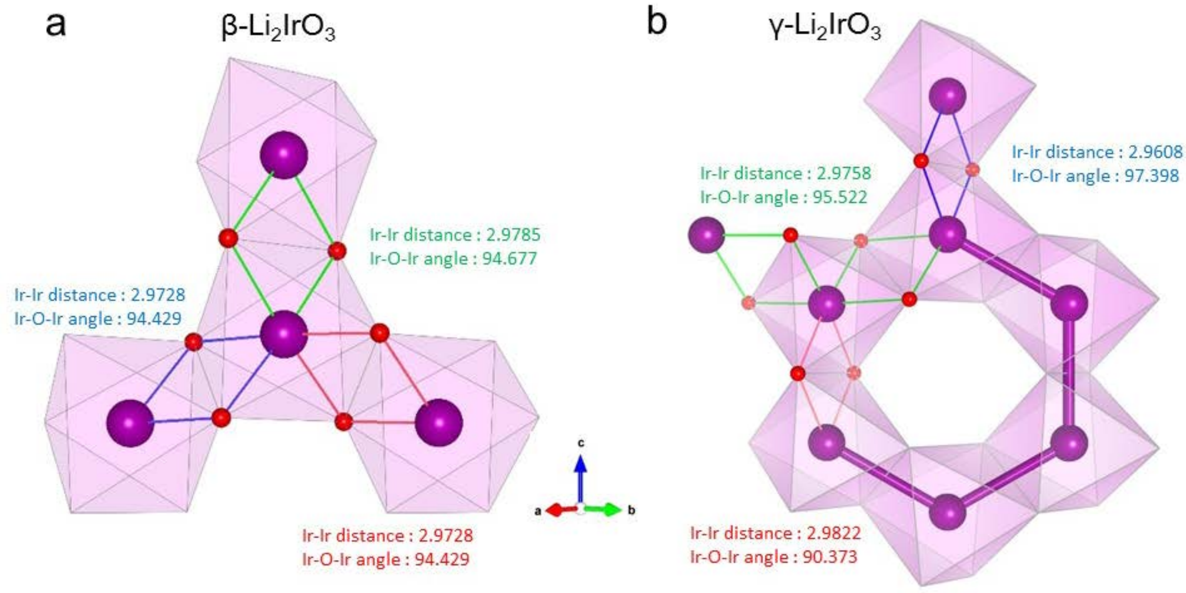

Supplementary Figure 2 | Network of IrO_6 octahedra and local bond geometries in the harmonic-honeycomb iridates β- and γ- Li_2IrO_3. Purple and red balls are iridium and oxygen atoms, respectively. See Supplementary Note 2 for detailed discussions.

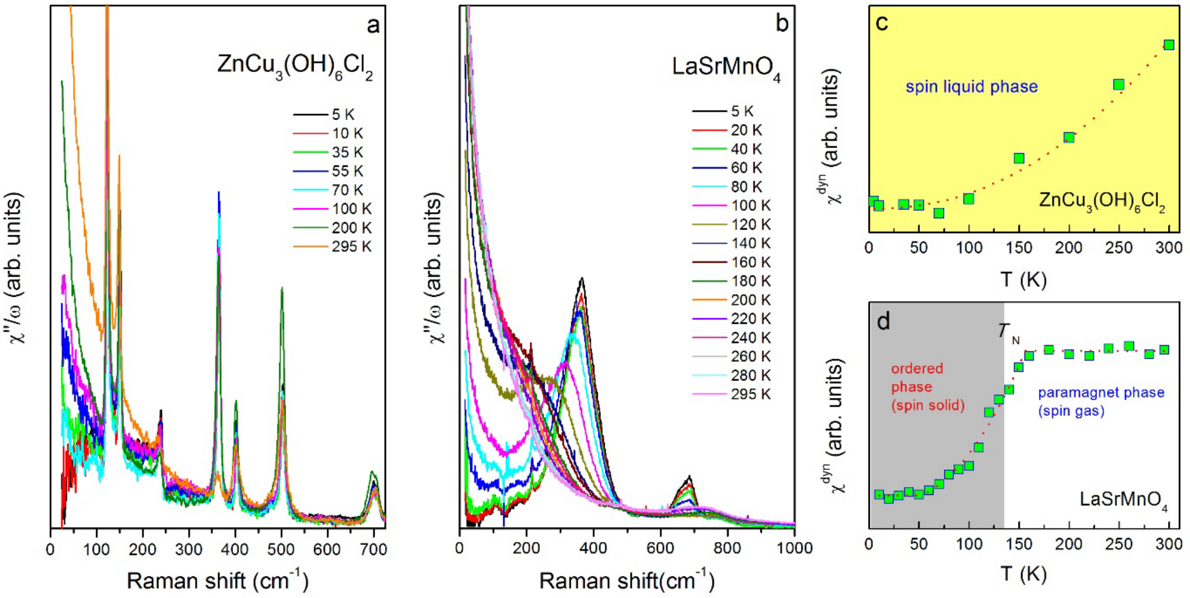

Supplementary Figure 3 | Comparison of the magnetic excitations between the kagome and the conventional antiferromagnets. (a) Temperature dependence of the Raman conductivity χ''(ω)/ω of the 2D kagome lattice $ZnCu_3(OH)_6Cl_2$. χ''(ω)/ω is calculated from the data taken from Ref. 7. (b) Temperature dependence of χ''(ω)/ω of the two-dimensional antiferromagnet $LaSrMnO_4$ with the antiferromagnetic ordering at T_N=133 K. χ''(ω)/ω is calculated from the data taken from Ref. 8. (c) Temperature dependence of the dynamic Raman susceptibility of $ZnCu_3(OH)_6Cl_2$. The red dashed line is a power-law fit to the data, $\chi^{dyn}(T)\sim T^{\alpha}$ with α=2.1. (d) Temperature dependence of the dynamic Raman susceptibility of $LaSrMnO_4$. The dashed lines are a guide to the eye.

Supplementary Table 1 | List of shell model parameters for the shell-shell and core-shell interactions. The interionic interactions employed for lattice dynamic calculations are discussed in Supplementary Note 1. This table lists the values of the shell model parameters for Born-Mayer-Buckingham and Morse potentials.

Born-Mayer-Buckingham potential							
Atom	$X(\|e\|)$	$Y(\|e\|)$	K(eV/Å)	Atomic pair	A(eV)	ρ(Å)	C(eVÅ^6)
Li	+1	0	0	Li-O	638.71	0.2906	0
O	+0.28	-1.5	29.5	O-O	22764.3	0.149	27.88
Morse potential							
Atom	$X(\|e\|)$	$Y(\|e\|)$	K(eV/Å)	Atomic pair	D(eV)	α(Å^{-1})	r_0(Å)
Ir	+1.66	0	0	Ir-O	3.66389	2.7245	2.026

Supplementary Table 2 | List of the observed and calculated frequencies of the A_g phonon modes in cm^{-1}. This table compares the experimental and simulated frequencies of seven A_g modes with normal mode displacement patterns. See Supplementary Fig. 1 and Supplementary Note 1 for detailed discussions.

Symmetry	Exp.	Cal.	Assignment
$A_g(1)$	185	193	Stretching vibrations of IrO_6 + Out-of-phase vibrations of Ir atom along the *z*-axis
$A_g(2)$	365	325	Bending vibrations of IrO_6 + Out-of-phase vibrations of Ir atom along the *z*-axis + Out-of-phase vibrations of Li(2) atom along the *z*-axis
$A_g(3)$	502	459	Bending vibrations of IrO_6 + Out-of-phase vibrations of Li(1) and Li(2) atoms along the *z*-axis
$A_g(4)$	527	547	Out-of-phase vibrations of Ir atom along the *z*-axis + Out-of-phase vibrations of Li(1) and Li(2) atoms along the *z*-axis
$A_g(5)$	582	594	Stretching vibrations of IrO_6
$A_g(6)$	602	605	Stretching vibrations of IrO_6
$A_g(7)$	643	651	Bending vibrations of IrO_6 + Out-of-phase vibrations of Ir atom along the *z*-axis + Out-of-phase vibrations of Li(1) and Li(2) atoms along the *z*-axis

Supplementary Note 1 | Phonon Spectra and Lattice Dynamical Calculations

β-Li_2IrO_3 possesses a *Fddd* structure[1]. The factor group analysis yields the total irreducible representation for the Raman-active modes $\Gamma = 7A_g\,(aa, bb, cc) + 8B_{1g}(ab) + 11B_{2g}(ac) + 10B_{3g}\,(bc)$. In order to assign the symmetries and eigenvectors to the observed optical phonon modes, we computed the Γ-point phonon modes by adopting shell-model lattice dynamical calculations implemented in the general utility lattice program (GULP) package[2].

Within the shell model, we treat an ion *Z* as a sum of a point core with charge *X* and a massless shell with charge *Y*, representing valence electrons. The ionic polarizability $\alpha = Y^2/K$ emanates from the interaction between the core and the shell, which is modeled as a harmonic oscillator with a force constant *K*. The interionic interactions between Li and O ions are described by a combination of long-range Coulomb potentials and short-range Born-Mayer-Buckingham potentials between ions *i* and *j* :

$$V_{BM}(r) = A_{ij}\exp(-r/\rho_{ij}) - C_{ij}/r^6, \qquad (1)$$

where A_{ij} and ρ_{ij} denote the strength and the range of the repulsive interaction, respectively, and C_{ij} describes an attractive part with the interatomic distance *r*. The interactions between Ir and O ions could be simulated by Morse potential:

$$V_M\,(r) = D_e[(1 - \exp(-\alpha(r - r_0)))^2 - 1], \qquad (2)$$

where D_e is the bond dissociation energy, *α* is the curvature of potential well and r_0 is the equilibrium distance between ions. Starting from well-documented data[3,4], the shell-model parameters are optimized to reach a reasonable agreement with experimental Raman data. The resulting shell-model parameters are summarized in Supplementary Table 1.

Supplementary Figure 1 shows the phonon Raman spectra of *β*-Li_2IrO_3 measured at $T = 300$ K in (*cc*) and (*ac*) polarization. In the (*cc*) scattering geometry we observe seven one-phonon excitations, which are in good agreement with the $7A_g$ mode predicted from the factor group analysis. In Supplementary Table 2 the observed peak frequencies are compared to the calculated ones. It is remarkable that there is no substantial discrepancy between the experimental and calculated frequencies in spite of the adopted simple potentials. Noticeably,

the A_g modes attain finite scattering intensities in the symmetry-forbidden (*ac*) polarization, indicative of the leakage of a selection rule. As single crystals of a size of 50 μm are tiny, there may exist a small mismatch between laboratory and crystal coordinate systems. In addition, the phonon bands of $500 - 700$ cm^{-1} seem to show no clear polarization dependence. This is largely because the B_{2g} and A_g phonon energies are nearly degenerated in a frequency range of $500 - 700$ cm^{-1}. The vibration pattern of the $A_g(1)$ Fano antiresonance is sketched in the inset of Figure 3a in the main text.

γ-Li_2IrO_3 has a *Cccm* structure[5]. According to the factor group analysis, the total irreducible representation for the Raman-active modes is given by $\Gamma = 14A_g(aa, bb, cc) + 15B_{1g}(ab) + 19B_{2g}(ac) + 18B_{3g}(bc)$. We observe the $14A_g$ modes in (*aa*) and (*cc*) polarizations, the $15B_{1g}$ modes in (*ab*) polarization, and the $18B_{2g}$ modes in (*ac*) polarization (see Figure 1c in the main text). We find good quantitative agreement the observed and calculated modes from the factor group analysis.

Supplementary Note 2 | Local bond geometry of β- and γ-Li_2IrO_3

β- and γ-Li_2IrO_3 are a member of the three-dimensional harmonic-honeycomb iridates, which comprise a tricoordinated network of edge-shared IrO_6 octahedra as sketched in Supplementary Figure 2. It is well known that Kitaev interactions are susceptible to trigonal distortions in the IrO_6 octahedra. These distortions give rise to deviations of the Ir-O bond lengths and the Ir-O-Ir bond angles from the ideal 90° Ir-O-Ir exchange path. β-Li_2IrO_3 has two different nearest-neighbor bonds with (i) the Ir-Ir distance 2.9785 Å and the Ir-O-Ir angle 94.677° and (ii) the Ir-Ir distance 2.9728 Å and the Ir-O-Ir angle 94.29°. As these two bonds are almost identical, the resulting Kitaev interactions of β-Li_2IrO_3 are near-isotropic with $J^x = J^y \approx J^z$. In contrast, its structural counterpart γ-Li_2IrO_3 shows sizable trigonal distortions, leading to appreciable deviations from the ideal structure. There are three distinct nearest-neighbor bonds: (i) the Ir-Ir distance 2.9822 Å and the Ir-O-Ir angle 90.373°, (ii) the Ir-Ir distance 2.9758° A and the Ir-O-Ir angle 95.522°, and (iii) the Ir-Ir distance 2.9608 Å and the Ir-O-Ir angle 97.398°. The resulting Kitaev exchanges become anisotropic with $J^x \neq J^y \neq J^z$, while distortion-induced and further neighbor interactions become stronger.

Supplementary Note 3 | Comparison of dynamic Raman susceptibility between conventional magnet and spin liquid material

In Supplementary Figure 3a,b we compare the Raman conductivity $\chi''(\omega)/\omega$ of the spin liquid material $ZnCu_3(OH)_6Cl_2$ and the conventional antiferromagnet $LaSrMnO_4$. In the former 2D kagome lattice with no magnetic ordering, $\chi''(\omega)/\omega$ exhibits a broad continuum centered around 120 cm^{-1} at $T = 5$ K[7]. The magnetic continuum is characteristic of fractionalized spinon excitations. With increasing temperature, $\chi''(\omega)/\omega$ develops progressively a pronounced peak at $\omega = 0$. In contrast, the 2D antiferromagnet $LaSrMnO_4$ ($3d^4$; $S = 2$) shows a well-defined maximum around 356 cm^{-1} as a two-magnon scattering below antiferromagnetic spin ordering at $T_N = 133$ K[8]. With increasing temperature through T_N its spectral weight broadens and shifts to lower energies and finally evolves to quasielastic scattering in the high temperatures. The quasielastic Raman response originates from an overdamping of short-range magnetic fluctuations.

We deduce the dynamic Raman susceptibility $\chi^{dyn}(T)$ of $ZnCu_3(OH)_6Cl_2$ and $LaSrMnO_4$ from the Raman conductivity as described in the Methods of the main text. The results are summarized in Supplementary Figure 3c,d. $ZnCu_3(OH)_6Cl_2$ displays a power-law increase of $\chi^{dyn}(T)$ with temperature, $\chi^{dyn}(T) \sim T^{2.1}$. This is in stark contrast to $\chi^{dyn}(T)$ of $LaSrMnO_4$, which strongly increases through T_N and shows saturation in a paramagnetic phase. The temperature independence of $\chi^{dyn}(T)$ is a defining feature of spin gas where spins are uncorrelated. In contrast, the power-law dependence of $\chi^{dyn}(T)$ in spin liquid suggests slowly decaying power-law spin correlations.

Supplementary Note 4 | Lattice anharmonicity

The temperature dependence of phonon self-energies is described in terms of the anharmonic model based on phonon-phonon decay processes to acoustic phonons[6]:

$$\omega(T) = \omega_0 + C[1 + 2/(e^x - 1)] + D[1 + 3/(e^y - 1) + 3/(e^y - 1)^2], \quad (3)$$

$$\Gamma(T) = \Gamma_0 + A[1 + 2/(e^x - 1)] + B[1 + 3/(e^y - 1) + 3/(e^y - 1)^2], \quad (4)$$

where $x = \hbar\omega_0/2k_BT$ and $y = \hbar\omega_0/3k_BT$ with A, B, C and D constants. ω_0 and Γ_0 are the frequency and the linewidth of an optical mode at zero temperature. The second and third terms originate from the decay of an optical phonon into three- and four-phonons due to anharmonicity, respectively. These processes lead to the respective quasi-linear and -quadratic temperature dependence at high temperatures. All phonon modes including the 24 meV Fano antiresonance display no noticeable anomalies in the temperature dependence of frequency and linewidth as discussed in the main text. This may be linked to the large unit cell and low crystal symmetry with a three-dimensional network of spins. In the very complex spin network, lattice vibrations involve the simultaneous modulations of different magnetic exchange paths and thus spin correlation effects on the phonon self-energy and linewidth are largely nullified.

Supplementary References

1. Takayama, T. *et al.* Hyperhoneycomb Iridate β−Li_2IrO_3 as a Platform for Kitaev Magnetism. *Phys. Rev. Lett.* **114**, 077202 (2015).

2. Gale, G. D. GULP: A computer program for the symmetry-adapted simulation of solids. *J. Chem. Soc., Faraday Trans.* **93**, 629-637 (1997).

3. Roos, J., Eames, C., Wood, S. M., Whiteside, A. & Islam, M. S. Unusual Mn coordination and redox chemistry in the high capacity borate cathode $Li_7Mn(BO_3)_3$. *Phys. Chem. Chem. Phys.* **17**, 22259-22265 (2015).

4. Sen, F. G. *et al.* Towards accurate prediction of catalytic activity in IrO_2 nanoclusters *via* first principles-based variable charge force field. *J. Mater. Chem. A* **3**, 18970-18982 (2015).

5. Modic, K. A. *et al.* Realization of a three-dimensional spin–anisotropic harmonic honeycomb iridate. *Nat. Commun.* **5**, 4203 (2014).

6. Balkanski, M., Wallis, R. F. & Haro, E. Anharmonic effects in light scattering due to optical phonons in silicon. *Phys. Rev. B* **28**, 1928-1934 (1983).

7. Wulferding, D. *et al.* Interplay of thermal and quantum spin fluctuations in the kagome lattice compound herbertsmithite. *Phys. Rev. B* **82**, 144412 (2010).

8. Choi, K.-Y. et al. Anomalous orbital dynamics in $LaSrMnO_4$ observed by Raman spectroscopy. *Phys. Rev. B* **77**, 064415 (2008).

9. Halperin, B. I. and Hohenberg, P. C. Hydrodynamic Theory of Spin Waves. *Phys. Rev.* **188**, 898-918 (1969).

10. Halley, J. W. Light Scattering as a Probe of Dynamical Critical Properties of Antiferromagnets. *Phys. Rev. Lett.* **41**, 1605-1608 (1978).
